# Advancing nuclear transfer cloning in zebrafish (*Danio rerio*) into a translational pathway using interdisciplinary tools

**DOI:** 10.1371/journal.pone.0312672

**Published:** 2024-12-30

**Authors:** Sarah Bodenstein, William Poulos, Fermin Jimenez, Michael Stout, Yue Liu, Zoltan M. Varga, Jose Cibelli, Terrence R. Tiersch

**Affiliations:** 1 Louisiana Sea Grant College Program, Louisiana State University, Baton Rouge, LA, United States of America; 2 Aquatic Germplasm and Genetic Resources Center, School of Renewable Natural Resources, Louisiana State University Agricultural Center, Baton Rouge, LA, United States of America; 3 College of Agriculture & Natural Resources, Michigan State University, East Lansing, MI, United States of America; 4 Zebrafish International Resource Center, University of Oregon, Eugene, OR, United States of America; Friedrich-Loeffler-Institute, GERMANY

## Abstract

The Zebrafish International Resource Center (ZIRC) is an NIH-funded national stock center and germplasm repository that maintains and distributes genetically modified and wild-type zebrafish (*Danio rerio*) lines to the biomedical research community. The ZIRC and its community would benefit from incorporating somatic cell nuclear transfer (SCNT) cloning which would allow the preservation of diploid genomes. The goal of this study was to advance a zebrafish SCNT cloning protocol into a reproducible community-level pathway by use of process mapping and simulation modeling approaches to address training requirements, process constraints, and quality management gaps. Training, for most steps in the SCNT protocol, could be completed within two months; however, steps that involved micromanipulation of eggs required more than four months of training. Dechorionation of embryos and egg micromanipulation were identified as major constraints because the processes were performed manually and required advanced operator manual skills. Chemical dechorionation and microfluidic devices to aid micromanipulation were identified as ways to eliminate these constraints. Finally, quality control steps to record the initial quality of collected germplasm were recommended to prevent production defects and harmonize the SCNT pathway across multiple facilities. By beginning to enhance the reproducibility of the SCNT cloning pathway, this technique can be implemented across zebrafish research facilities and facilities that work with other biomedical models.

## Introduction

All research protocols begin as “benchtop-level” protocols, meaning the process can be reproduced successfully in the facility where it was created. There are often challenges, however, with translating these protocols to other facilities or scaling up protocols to meet commercial-scale production needs. The protocol for a novel cloning technique, somatic cell nuclear transfer (SCNT), in zebrafish (*Danio rerio*) currently falls into this benchtop-level category. There is significant potential application for this protocol across the zebrafish research community, particularly at the Zebrafish International Resource Center (ZIRC). The ZIRC is a central genetic repository that collects, maintains, and distributes genetically modified and wild-type zebrafish lines, research materials, and diagnostic health services to the biomedical research community. Currently, ZIRC houses and provides 12,823 genetically modified lines (representing 46,127 distinct alleles) and imports hundreds of new lines each year. These lines are banked as cryopreserved sperm samples that are thawed upon request for *in vitro* fertilization with wild-type (AB) eggs and subsequently distributed as batches of heterozygous larvae or, after rearing, as genetically identified adult heterozygotes. However, the original AB DNA sequence was determined years ago in select individuals [1]. Random genetic drift can modify the genome sequences of the live population that is needed to provide eggs for sperm reactivation, whereas the cryopreserved fish lines remain unchanged [2, 3]. Thus, only the haploid genome is preserved and reactivated lines distributed by ZIRC may not have the exact same genomic composition as the acquired and stored fish. The ZIRC also imports lines with varying or absent information about their health status. While ZIRC employs strict biosafety measures and expends great effort on the health monitoring of its colony, elimination of diagnosed pathogens in acquired lines is an arduous process. This process can include raising reactivated generations of a line in the quarantine room for additional rounds of observation, diagnosis, and selection of healthy individuals before they are allowed access to the main fish facility. This process is extremely time consuming and laborious.

One solution is somatic cell nuclear transfer (SCNT), an assisted reproductive technique widely implemented in domestic and exotic animal species. It entails the transplantation of the nucleus of a somatic cell into the cytosol of an enucleated matured oocyte of the same or closely related species [4]. Its current uses include the horizontal dissemination of genetically superior animals and the creation of animal models for human disease, sports, and companion animals. Efforts to recover threatened and extinct species also rely on SCNT [5]. John Gurdon first reported cloning animals using non-embryonic cells in *Xenopus laevis* [6]. The first report of zebrafish cloning was published almost 20 years later utilizing artificial diploidization of gynogenetic haploid embryos (Streisinger et a., 1981). Cloning with somatic zebrafish cells was not achieved until 2002 [7].

The SCNT method is used as a model system to study the process of cellular de-differentiation in two species, bovine and zebrafish [8, 9]. It is worth mentioning that, given its unique reproductive system, zebrafish presents significant advantages over mammalian species. Organogenesis begins less than 24 hours after fertilization, offering rapid embryonic development and straightforward observation due to the optical transparency of both chorion and embryo [10]. There is no limitation on the number of unfertilized oocytes needed to perform the procedure because females can produce several hundred eggs in a single spawning event. Furthermore, zebrafish reproduce year-round under standard laboratory conditions, and embryos develop outside the female under simple culture conditions. Some mammalian offspring produced with SCNT cloning have displayed placental, heart, kidney and lung defects, although the majority go on to be healthy and have a normal lifespan. In zebrafish, however, the rate of abnormalities in cloned fish is low [11]. A streamlined protocol for zebrafish SCNT has been developed with potential for use in reconstitution of fish lines cryopreserved as diploid cells. The Cibelli laboratory uses SCNT as a model system to study the process of cellular de-differentiation in two species, bovine and zebrafish. Detailed protocols for both techniques can be found in our publications [8].

Based on a survey in 2023, there is significant interest in the zebrafish research community for ZIRC to incorporate SCNT cloning services (Z. Varga, pers. comm.). More than 55% of respondents (n = 593) favored cryopreservation services by ZIRC, including conventional cryopreservation and SCNT, if the preserved material was made publicly accessible. While a SCNT cloning protocol for zebrafish has been developed and refined at the benchtop-level, transferring this protocol to other facilities, such as ZIRC, presents a problem. Research protocols contain limitations, often due to a lack of quality management steps, that makes their transfer difficult with respect to obtaining consistent results. The field of industrial engineering provides tools that can delineate protocols so they can be fully understood while identifying gaps where quality management steps can be incorporated. Industrial engineering is primarily used in the manufacturing sector to identify and eliminate production inefficiencies (waste) with the goal of improving production capabilities [12]. Process maps and discrete-event simulation models are a commonly used tools that outlines the flow of materials and information in a protocol to identify bottlenecks (constraints) and wastes [13, 14]. Previous studies have employed these tools to model high-throughput or commercial-scale cryopreservation pathways either to increase efficiency or to establish new protocols in existing systems [15–17]. Genetic resource centers focus on acquiring, propagating, and redistributing accurately identified genetic strains and become the long-term custodians of these genetic resources for their research communities. Therefore, they place particular emphasis on both quality management and the efficient implementation of new technologies, and could benefit from using industrial engineering approaches. However, the research community is also concerned with experimental rigor and reproducibility of operator performance and training [18].

The goal of this study was to use process mapping to advance a nuclear transfer-cloning protocol in zebrafish into a reproducible and generalizable pathway. The objectives were to: 1) define the steps of the current cloning protocol and outline steps in a process map; 2) identify the constraints of the protocol and the level of training required to complete different steps; 3) provide recommendations to eliminate the constraints and advance a robust protocol into a reproducible pathway, capable of being implemented at ZIRC; 4) modify the pathway for use at other facilities, such as research laboratories or genetic stock centers, and 5) describe future steps to ensure generalizability and broader applications.

## Materials and methods

### Process mapping of the cryopreservation pathway

All the steps of the somatic cell nuclear transfer (SCNT) cloning protocol were identified and defined during six discussions on Zoom with Cibelli laboratory members. While this SCNT protocol has been published [4, 19, 20], the following description focuses on the flow of materials and information through the process and is designed to avoid any logical or temporal gaps. This study is based on published protocols that report an efficiency of 2% for cloned fish production using SCNT [21]. Steps were outlined in a process map, a tool commonly used in industrial engineering to illustrate and understand a specific process [13, 22]. This study focused on three main stages of the SCNT protocol: Cell Culture (Steps 1–7), Egg Preparation (Steps 8–14), and Cloning (Steps 15–30). When steps were defined in this study, previous work and data were repurposed by Cibelli laboratory members, who provided consensus estimates (based on past experience) of step definitions and how long each step took. Due to this, no animal husbandry or experimentation was performed directly by authors at Louisiana State University or University of Oregon. Authors in the Cibelli laboratory at Michigan State University obtained IACUC permits for the work (see Permits and Approvals section). In addition, no protected species were sampled, and all work was performed in university-owned laboratory facilities.

### Cell culture stage (Steps 1–7)

Steps 1–7 were conducted at least 2 weeks before egg preparation and cloning. In **Step 1**, zebrafish are placed in breeding tanks (1.7-L Sloping Breeding Tank, Tecniplast) and allowed to spawn up to 2 hours. Following courtship behavior and once enough fertilized eggs are collected (approximately 200), embryos are collected and placed into embryo media solution (S1 File) and 0.5mg/L methylene blue (an anti-fungal agent). In **Step 2**, embryos are disinfected using 0.5% bleach for 30 sec, 70% ethanol for 20 sec, and rinsed three times with Dulbecco’s phosphate-buffered saline (DPBS, Gibco—cat#D8537-500ML). Embryos are stored overnight (18 hours) in an incubator at 28.5°C to allow for development. At 2–4 hours post fertilization (hpf), embryo survival is assessed by confirming cell division and chorion expansion. Unfertilized or morphologically abnormal eggs are culled before proceeding to the next step.

**Step 3** begins if embryo survival is greater than 80% and 24 hpf embryos appear morphologically normal. Approximately 30–60 embryos (depending on the number of operators performing nuclear transfer) are selected for dechorionation. Embryos are removed from chorions and placed in a 168 mg/L Tricaine—-phosphate-buffered saline solution to immobilize them (Ethyl 3-aminobenzoate methanesulfonate—Sigma-Aldrich cat# E10521). The embryos are placed in a drop of DPBS (circa 100–200 μL) and the chorions are removed manually with two 27G hypodermic needles (BD, cat# BD 305109). One needle is used to anchor the chorion and the other is used to slice it. In **Step 4**, immobilized embryos are disinfected with 30mg/L bleach for 30 sec, 70% ethanol for 20 sec, and rinsed three times with DPBS. In **Step 5**, each embryo is placed into a dish (Nunc Cell Culture/Petri dishes—Cat# 153066) with prepared DNAC culture media (S1 File). Embryo tails are removed and saved for further dissociation and the remaining portion of the embryo is discarded.

In **Step 6**, the tips of embryo tails are cut into 300–400 μM pieces with fine-tip surgical scissor or 27G-needles and each piece is transferred to a Nunc™ MicroWell™ 96-Well plate (cat# 167008) coated with 5 mL of a membrane matrix (Corning® Matrigel®, cat# 356234). After Step 6, the cells should be passaged 1–2 times, taking a minimum of two weeks (**Step 7)**. Cells are detached from the dish and centrifuged at 3,500 RPM for 5 minutes. The supernatant is removed, and cells are resuspended in a Sigma 2.0-mL cryogenic vial (Sigma, cat#SIAL0659) containing at least 250 μL of DNAC freezing media depending on cell density (S1 File). Vials are placed in a freezing container (Mr. Frosty™ Freezing Container ThermoFisher #5100–0001) in a -80°C freezer for at least 24 hours and are moved to a cryogenic storage tank for long-term, liquid-phase storage.

### Egg preparation stage (Steps 8–14)

**Step 8**, the beginning of the *Egg Preparation* stage, consisted of placing one male and one female zebrafish on either side of a mesh screen in a breeding tank. The fish are left over night for 15–16 hours. In **Step 9**, the mesh screen is removed, and the male and female are placed together in a fresh tank for 1 hour to allow for natural breeding. Males and females are separated as soon as natural breeding occurs. Egg quality is assessed visually and if the fertilization rate is less than 80% or embryo morphology is abnormal, embryos are discarded, and the procedure is ended and must be repeated. In **Step 10**, females that were observed to produce high-quality embryos (fertilization rate was greater than 80% and all eggs appear morphologically normal) in Step 9 are anesthetized using 0.02% Tricaine solution to prepare for egg collection.

In **Step 11**, eggs are collected separately from the females anesthetized in Step 10 using standard laboratory techniques (adapted from [23]). Eggs are placed into a drop of chinook salmon ovarian fluid (CSOF) to prevent activation. This allows for 3 hours of manipulation time without a significant decrease in fertilization potential (adapted from [23]). After all eggs are collected, in **Step 12**, they are transferred from the original drop of CSOF to a new drop to rinse away any urine or feces. In **Step 13**, egg nuclei are stained with 0.2% Hoechst nuclear stain (Hoechst 33342, Trihydrochloride, Trihydrate, 100 mg—ThermoFisher, Cat#H1399) while in the CSOF solution. After a 30-minute waiting period, in **Step 14**, eggs are rinsed three times in CSOF to remove the remaining nuclear stain.

### Cloning stage (Steps 15–30)

**Step 15** involves thawing cells cryopreserved in Step 7. Cells are thawed in a water bath (Thermo, cat#TSGP10) at 28.5°C before resuspension in a 2X volume of fetal bovine serum (Fetal Bovine Serum—Thermo Fisher Scientific, cat#10439) [24]. Cells are centrifuged for 5 minutes at 3,500 RPM and the supernatant is removed. In **Step 16**, cells are resuspended in 2% Polyvinylpyrrolidone in LHC media in preparation for plating (Polyvinylpyrrolidone (PVP)—Sigma-Aldrich cat#PVP40, Laboratory of Human Carcinogenesis (LHC) basal medium—Thermo Fisher Scientific, cat#12677–027). On the manipulation dish, in **Step 17**, two drops of 5% PVP-CSOF are plated. The PVP-CSOF drops function as the manipulation media for the eggs. Three drops of 4–5 μL each of the cells suspended in 2% PVP-LHC are plated using a P20 pipette on a dish next to the two drops of 5%PVP-CSOF. The manipulation dish is flooded with mineral oil (Mineral Oil—Sigma-Aldrich (cat#330779-1L) to prevent evaporation and to facilitate the manipulation of drops.

**Fig 1:**
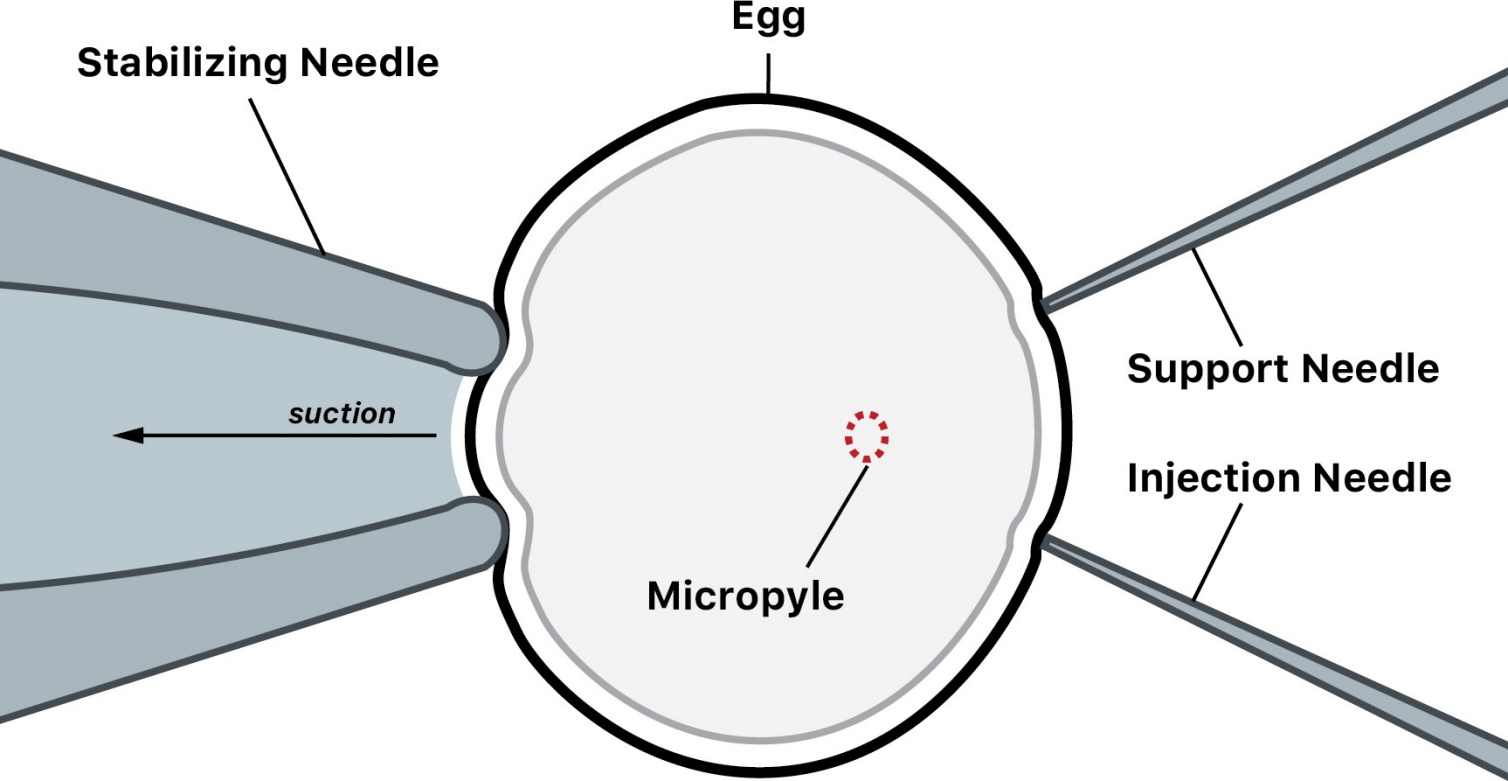
Setup for zebrafish egg manipulation. A diagram displaying how zebrafish eggs are manipulated during SCNT cloning in Steps 20–23. Eggs are held in place with a stabilizing needle connected to the Celltram®4r air using gentle suction and are rotated with two manipulation needles (one for support and the injection needle). The injection needle is connected to the Celltram®4r vario to inject the cell and the manipulation needle is connected to a needle holder. The micropyle is first positioned to face directly towards the lens of the microscope (on the Z-axis) on the manipulation dish for enucleation and then rotated 90 degrees and positioned to face the 3 o’clock position (on the X-axis). This diagram is based on figures from Siripattarapravat et al. (2014).

In **Step 18**, the rest of the equipment and supplies needed for the *Cloning* stage are prepared. This includes an inverted-stage microscope, two microcapillaries connected to micro-injectors for manipulation of eggs, a Celltram®4r air manipulator (Eppendorf 5176 000.017), a Celltram®4r vario for pressure control and microinjections (Eppendorf 5176 000.025), as well as the laser and associated software (XYClone, Hamilton-Thorne). One manipulation capillary is connected to the Celltram®4r vario and used to inject the egg as well as position it in the dish. The other manipulation capillary is attached to a mechanical micromanipulator and used to hold and position the egg in place. The stabilizing needle is connected to the Celltram®4r air manipulator which provides gentle suction to pick up and hold the egg without completely restricting movement (Fig 1).

In **Step 19**, eggs are rinsed in 5% PVP-CSOF to minimize dilution of manipulation drops and the desired number of eggs (generally 4–5) are placed into a single drop on a manipulation dish. Steps 20–25, where the somatic cell nuclear transfer (SCNT) takes place, involves repetitive precision work performed on single eggs until all eggs are processed. Depending on numbers, eggs can be divided into batches on each dish. In general, each operator can process six batches of five eggs (30 eggs total) in Steps 20–25. The remaining subset of eggs are set aside until Step 27. Each batch is processed individually and upon completion of Step 24, the next batch begins processing.

In **Step 20**, a manipulation dish is placed under the microscope, the egg micropyle is located, and the egg is positioned using the manipulation capillaries. The micropyle is a cone-shaped channel composed of the micropylar groove (circa 8 μm in diameter) and the micropylar pit (circa 3 μm in diameter) through which sperm enter the egg [19, 20]. The micropyle is positioned to face directly towards the bottom of the manipulation dish, as close to the microscope objective as possible. The egg’s nucleus is moved into focus and aligned to the laser’s crosshair using a 350 nm UV excitation filter. In Step 13 before, each egg was treated with a DNA stain (Hoechst nuclear stain) that can pass through cell membranes. When exposed to UV light, the stain becomes excited in the nucleus, making it visible.

In **Step 21**, the egg DNA is ablated using the XYClone Hamilton-Thorne laser with two consecutive 400 μs exposures. In **Step 22**, the micropyle is realigned, facing the 3 o’clock position (rotated clockwise 90 degrees). In **Step 23**, a somatic cell is placed on the same manipulation dish and aspirated into the injection microcapillary. The operator confirms that the cell membrane is broken to allow the nucleus to enter the donor egg. The microinjection needle is aligned with the micropyle of the enucleated egg and the nucleus is injected into its cytoplasm. In **Step 24** all eggs are placed into CSOF and allowed to rest for 15 minutes after steps 21–23 are completed for each egg on a single dish (a batch). In **Step 25**, eggs are activated by placing them in 1% HBSS (9.7 g/L) in double-distilled water supplemented with 5% Bovine Serum Albumen (BSA, Sigma-Aldrich, No. 9048-46-8c). Eggs are placed into individual wells of a 96-well plate to allow for development. If additional eggs remain, a new batch is processed through Steps 20–25 until all eggs are processed.

In **Step 26**, male zebrafish are anesthetized in 168 mg/L tricaine solution to minimize risk of injury during milt extraction (adapted from [23]). The flank of a male is gently squeezed and milt is instantly collected into a 5-μL glass micropipette by capillary force [19]. Milt collected from 2–3 males is pooled and suspended in 9.7 g/L HBSS. In **Step 27**, the subset of eggs set aside before Step 20 (i.e., eggs that are not processed for somatic cell nuclear transfer), undergo *in-vitro* fertilization by combining the milt collected in Step 26 and embryo media solution (S1 File) (adapted from [23]). This is done to evaluate the fertilization potential of eggs that were processed for SCNT embryos. Finally, in **Step 28**, embryos are placed into individual wells of a 96-well plate. After 18–24 hours, if fertilization success is less than 70% all fertilized embryos as well as embryos that underwent somatic cell nuclear transfer are discarded and the process is terminated. If fertilization success is greater than 70%, dead or morphologically abnormal embryos are culled in **Step 29**, and SCNT embryos are reared to adulthood over the next four months in **Step 30**.

### Operator experience

**Fig 2:**
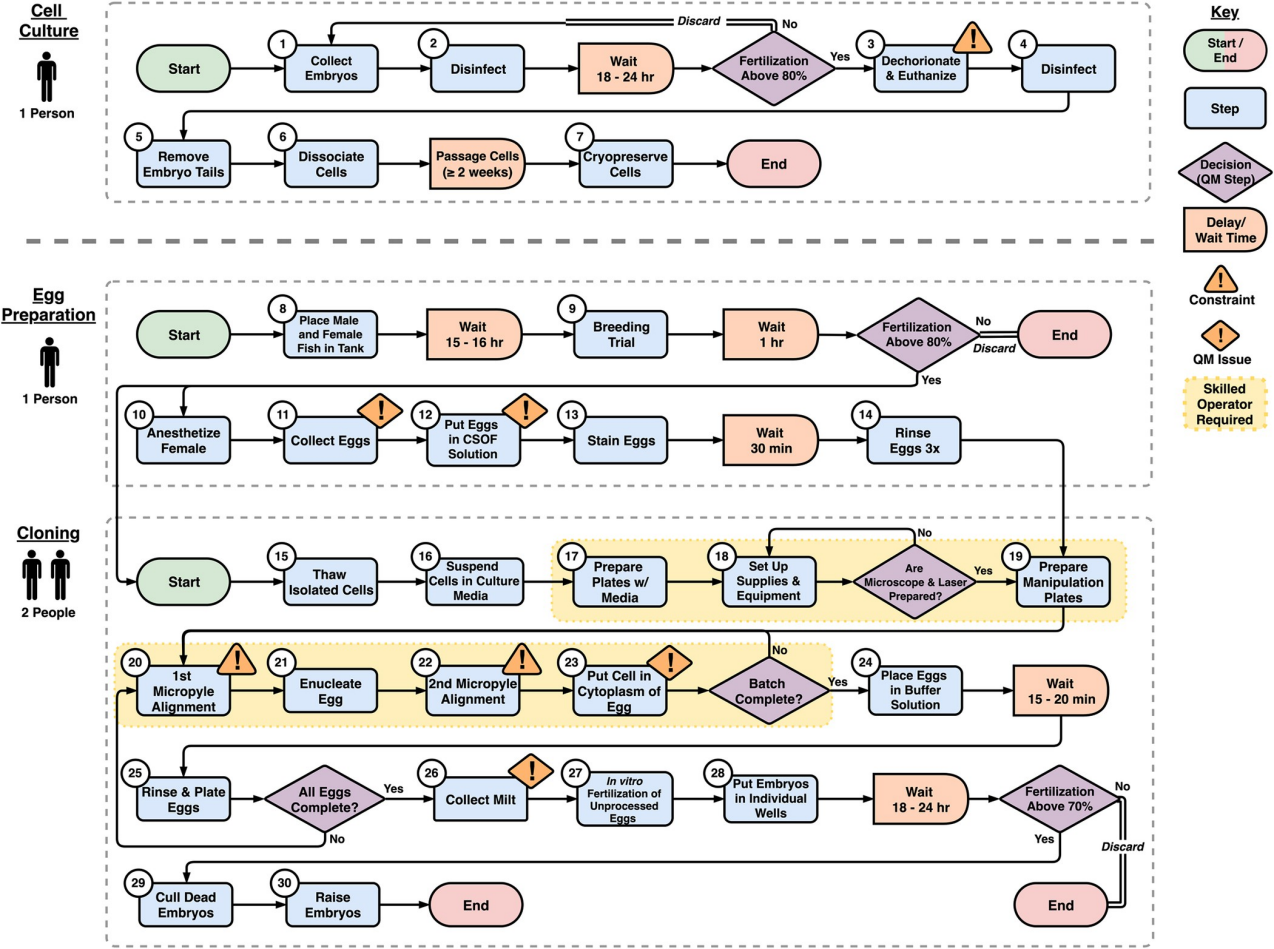
Process flow map of the somatic cell nuclear transfer (SCNT) cloning protocol. The protocol was divided into three stages: the Cell Culture, Egg Preparation, and Cloning stages. Oval shapes represent the start and end steps in the protocol; rectangles indicate steps in the protocol; rectangles with one curved side indicate a delay or wait time; large diamonds indicate quality assurance (QA) steps; and small triangles indicate steps that are constraints to the system. Steps with a colored background must be performed by skilled operators who have received specialized SCNT training. Single-line arrows indicate the flow of material, whereas double lines indicate that material was removed from the system. Steps in this map can be customized for any process.

The level of skill and training required by operators to complete each step (Fig 2) was determined via discussions with current Cibelli laboratory members (pers. comm., W. Poulos and F. Jimenez). An estimate (based on experience) of the level of training required, in weeks, for each step was provided. In addition, the suggested level of operator skill required for each step in the process was provided to determine which laboratory member should perform what steps. The skill levels were assigned into two categories. The first category was a *Part-Time* staff member, such as an undergraduate student worker or short-term laboratory member, who would have limited time in the laboratory each day and be a member for less than 2 years. The second category was a *Long-Term* member, such as a full-time staff member, principal investigator, or graduate student. A *Long-Term* member would spend most of the day in the laboratory and be a member for longer than 2 years.

A discrete-event simulation model was also created to evaluate the effect of operator experience on production capacity. The simulation model encompassed the steps in the SCNT process that were highlighted as requiring a skilled operator (Steps 17–23, Fig 2). To construct the model, an operator at the Cibelli laboratory recorded the required time to complete Steps 17–23. Each step had at least three replicate times recorded (Table 1). Time distributions were generated in MATLAB (v23.2.0. 2409890, The MathWorks, Natick, MA) for each step using replicate times. Six distributions were tested against time data for each step using the Kolmogorov–Smirnov goodness of fit test: exponential, gamma, lognormal, normal, poisson, and Weibull. The distribution with the best fit was selected to represent the data. For steps with less than five time replicates, a normal distribution was generated with available data.

**Table 1:** Estimated time to complete each step in the SCNT cloning protocol. *Values inside parentheses indicate times for “expert” operators with more experience*. *The times in this table are based upon operators processing six batches of five eggs*, *30 eggs total*.

Step	Description	Time (min)	Unit
**1**	*Collect Embryos*	180	total time for step
**2**	*Disinfect*	5	total time for step
		**Wait 18–24 hours**	
**3**	*Dechorionate & Euthanize*	2 (0.25)	per embryo
**4**	*Disinfect*	5	total time for step
**5**	*Remove Embryo Tails*	20	total time for step
**6**	*Dissociate Cells*	20	total time for step
		**Wait ≥ 2 weeks**	
**7**	*Cryopreserve Cells*	90–120	total time for step
**8**	*Prepare Breeding Trial*	30	total time for step
		**Wait 15–16 hours**	
**9**	*Breeding Trial*	30	total time for step
		**Wait 1 hour**	
**10**	*Anesthetize Females*	2	per fish
**11**	*Collect Eggs*	1–2	per fish
**12**	*CSOF Solution*	2	total time for step
**13**	*Stain Eggs*	30	total time for step
		**Wait 30 minutes**	
**14**	*Rinse Eggs*	5	total time for step
**15**	*Thaw Isolated Cells*	10	total time for step
**16**	*Suspend Cells in Media*	5	total time for step
**17**	*Prepare Dishes w/ Media*	10	total time for step
**18**	*Set up Equipment*	15	total time for step
**19**	*Prep*. *Manipulation Dish*	2	per batch
**20**	*1*^*st*^ *Micropyle Alignment*	5–10 (2)	per egg
**21**	*Enucleate Egg*	0.5	per egg
**22**	*2*^*nd*^ *Micropyle Alignment*	5–10 (1)	per egg
**23**	*Insert Cell in Egg*	1	per egg
**24**	*Eggs in Buffer Solution*	20	per batch
		**Wait 15–20 minutes**	
**25**	*Rinse & Plate Eggs*	10	total time for step
**26**	*Collect Milt*	15	per male
**27**	*In vitro Fertilization*	5	total time for step
**28**	*Plate Embryos*	10	total time for step
		**Wait 18–24 hours**	
**29**	*Cull Dead Embryos*	15	total time for step
**30**	*Raise Embryos*	4 months	total time for step

The time data collected and distributions generated were representative of an expert operator, with over 3 years of training and practice in SCNT cloning with zebrafish (W. Poulos pers. comm.). To generate time data for operators of different skill levels the Westinghouse System of rating operator skill was employed [25]. The Westinghouse System rates operators in four categories: skill, effort, conditions, and consistency. Skill was the operator’s proficiency at following given methods and effort was the operator’s willingness to expend energy in effective work. Conditions were the non-human factors that affected productivity and consistency was the potential for operator variability [25]. The operator whose times were observed in this study was rated to be an expert operator according to person statements and level of experience (W. Poulos pers. comm.). The Westinghouse ratings given to the expert operator assumed “excellent” ratings for Skill, Effort, and Consistency, and a “good” rating for Conditions (Table 2). The average operator was given neutral ratings (ratings of zero or “average”) for all four categories. The beginner operator ratings assumed a “poor” rating for Skill, a “fair” rating Consistency, and the same ratings for Effort and Conditions as the average operator.

**Table 2:** The rating level and factor for the four elements of the Westinghouse system for rating operator skill level [25]. *The four elements are skill*, *effort*, *conditions*, *and consistency*. *Ratings were provided for each of three operator skill levels*: *beginner*, *average*, *and expert*.

Operator Skill Level	Skill		Effort		Conditions		Consistency		Total
	*Rating Level*	*Rating Factor*	*Rating Level*	*Rating Factor*	*Rating Level*	*Rating Factor*	*Rating Level*	*Rating Factor*	
***Beginner***	F	-0.19	D	0	D	0	E	-0.02	***0*.*79***
***Average***	D	0	D	0	D	0	D	0	***1*.*00***
***Expert***	B1	0.11	A	0.125	C	0.02	A	0.04	***1*.*30***

After total operator skill rating was calculated following the Westinghouse rating table [25], adjust process times for each step based on operator skill were calculated using the following equation:

$${Step}_{X}={Step}_{Exp.}+{[Step}_{Exp.}\times ({Rating}_{Exp.}-{Rating}_{X})]$$

where *Step_X_* was the adjusted processing time for a step with an average or beginner operator, *Step_Exp._* was the average processing time for a step based on recorded times for an expert operator, *Rating_Exp._* was the total operator skill rating for an expert operator, and *Rating_X_* was the total operator skill rating for an average or beginner operator. The *Step_Exp._* average processing time was based on 10 replicate times generated using the time distributions for each step. The time distributions for each step generated for the expert operator were inserted into the software Simio (v15.240, Simio LLC, Sewickley, PA) to create a simulation model, the Expert Model. In addition, the adjusted processing times of each step for the average and beginner operators were inserted into Simio to create the Average and Beginner Models. Each of the three models was run 10 times to generate data for the total, required processing time based on the number of embryos. Statistical analyses were performed in RStudio (version 2023.06.1, R Core Team, 2023). A linear regression was used to determine the effect on processing time of operator skill level (beginner, average, or expert) and the number of manipulation dishes to be processed.

Each model contained a set of logic rules that that allowed the model to represent real-world conditions as closely as possible. The models contained the following logic rules:

Logic Rule 1: All steps in the models required an operator to begin processing. The steps had “progress triggers” which before processing, would “seize” an operator and “release” the operator after processing was completed (terminology in quotations was used within the modeling software).

Logic Rule 2: Each manipulation dish prepared in Step 17 contained five embryos to be processed.

Logic Rule 3: All manipulation dishes with embryos had to pass through the Step 19 “server” (i.e., finished processing in Step 19) before any embryos could begin processing in Step 20.

### Protocol constraints and quality management

Constraints, also called bottlenecks, are points of operational congestion in a process that stop or severely delay it [26, 27]. In the present study, constraints in the SCNT cloning protocol were identified by discussions with Cibelli laboratory members, who provided insight into the nature of congestion. In addition, the estimated time of each step provided by laboratory members was reviewed and discussed to detect any previously unidentified constraints. Solutions to alleviate constraints and make the protocol applicable to a wider range of facilities were discussed.

Places in the protocol where quality management steps could be inserted were also discussed. Integrating quality management (QM) steps into a protocol ensures the process is reproducible across multiple facilities that may use different equipment options [i.e., harmonization, 28, 29]. Quality management also considers production demands, establishing a process that can operate at multiple scales of production. Two components of quality management are quality assurance (QA) and quality control (QC). Quality assurance steps are “process oriented” activities that prevent defects or errors from occurring with the material being handled (Torres et al. 2016). An example of a QA step would be to calibrate a piece of equipment before use to ensure it performs properly and to prevent defects, such as calibrating a pH meter. Quality control steps are “material oriented” and identify defects in the material being handled (Torres et al. 2016). An example of a QC step would be checking the quality of a product halfway through the production process, such as measuring fertilization rates to assess egg quality. If quality standards are met the product can continue through the process, if not the product is discarded minimizing further wasted effort.

## Results

### Process mapping of the cryopreservation pathway

A comprehensive process map was developed based on descriptions of the steps in the SCNT cloning protocol by Cibelli laboratory members. In total, 30 steps separated into three stages (Cell Culture, Egg Preparation, and Cloning) were identified and described (Fig 2). In addition, three constraints (system bottlenecks) in Steps 3, 20, and 22, as well as three quality management gaps in Steps 11, 12, and 26 were identified. Descriptions of identified constraints and quality management gaps are detailed in Section 3.3.

### Operator experience

For Steps 1–16 and 25–30, all processes can be completed by a *Part-Time* laboratory member, such as an undergraduate student worker. Training to complete the activities encompassed in Step 1–16 and 25–30 would require circa 8 weeks. Steps 17–23, must be completed by *Long-Term* laboratory members, such as a principal investigator, graduate student, or full-time staff member. Operators participating in Steps 17–23 require specialized SCNT training and practice that requires between 16–24 weeks (4–6 months).

Through discussions with personnel in the Cibelli laboratory and using the Westinghouse System for rating operator skill, the operator observed in this study was determined to be at the expert level. Total skill ratings for expert, average, and beginner operators were calculated (Table 2). The skill rating of the expert operator was 30% higher than the average operator and 65% higher than the beginner operator. After skill ratings were applied to calculate adjusted processing times for Steps 17–23, the times for an average operator were on average 30% higher than the times for an expert operator. The processing times for a beginner operator were on average 50% higher than for an expert operator and 15% higher than for an average operator (Table 3).

**Table 3:** The processing time, in minutes, required for Step 17–23 in the SCNT protocol for operators of each skill level: Beginner, average, and expert. *Half-widths (HW*, *distance from the confidence limits to the mean for two-sided intervals) are displayed next to processing times*.

Step	Processing Time (min ± HW)			
	*Beginner Operator*	*Average Operator*	*Expert Operator*	*Unit*
***17***	2.8 ± 0.1	2.4 ± 0.1	1.9 ± 0.1	*per dish*
***18***	2.2 ± 0.0	1.9 ± 0.0	1.5 ± 0.0	*per dish*
***19***	0.8 ± 0.1	0.7 ± 0.1	0.5 ± 0.1	*per embryo*
***20***	0.7 ± 0.1	0.6 ± 0.1	0.5 ± 0.1	*per embryo*
***21***	0.3 ± 0.1	0.3 ± 0.1	0.2 ± 0.1	*per embryo*
***22***	0.6 ± 0.1	0.5 ± 0.1	0.4 ± 0.1	*per embryo*
***23***	1.4 ± 0.1	1.2 ± 0.1	0.9 ± 0.0	*per embryo*

**Fig 3:**
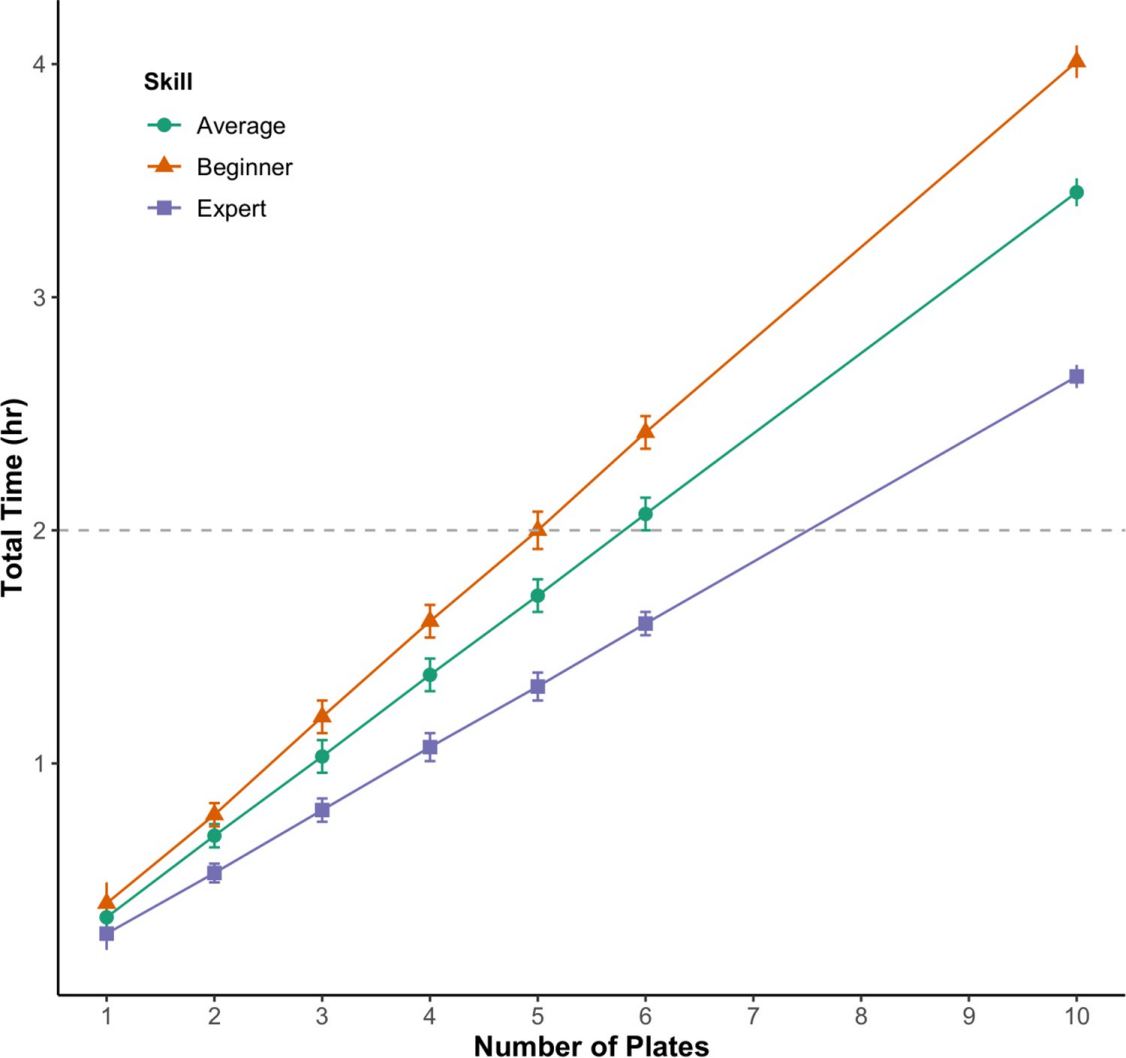
Plot of the total processing times. The plot shows the times required to process different number of manipulation dishes (each with five embryos) for operators of average, beginner, and expert skill levels. Bars on each point represent half-widths (distance from the confidence limits to the mean for two-sided intervals). The horizontal, dashed line represents the maximum time embryos should be left on dishes before the quality begins to decline (two hours).

When the processing times for the steps were inserted into three simulation models (one for each operator skill level), the total processing times were calculated based on the number of manipulation dishes (i.e., the number embryos). There was a significant interaction between operator skill level and number of dishes on total processing time (general linear model, *P* < 0.01, for all comparisons). The beginner operator had the steepest slope (a 24-min increase for each additional dish), meaning an increase in the number of dishes had the largest effect on a beginner operator (Fig 3). The average operator saw a 21-min increase for each additional dish, while an increase in the number of dishes had the smallest effect on an expert operator; a 16-min increase for each additional dish.

### Protocol constraints and quality management

The constraints identified in the SCNT cloning protocol occurred at Steps 3, 20, and 22. In Step 3, zebrafish embryos were dechorionated and euthanized. The process of dechorionation was manual, meaning that two hypodermic needles were used to pull the chorion from each embryo, processed individually by an operator. The estimated time to dechorionate each embryo was 2 minutes when a single operator processed approximately 30 embryos (Table 1).

Another major bottleneck occurred in Steps 20 and 22. In each step, the micropyle of the egg needed to be aligned to enucleate the egg (first alignment in Step 20) and to inject the contents of a somatic cell into the egg (second alignment in Step 22). Laboratory members attested to the difficulty in aligning the egg due to the extensive training required to recognize the micropyle and be able to position the egg with manipulation capillaries. The egg manipulation instruments were part of the reason months of training were required for proper manipulation. A stabilizing needle is used to gently secure one side of the egg, allowing it to be stabilized but not completely inhibiting rotation (Fig 1). This method, however, could result in the egg being damaged due to excessive suction force. Identifying the micropyle efficiently also took months of training. Operators with less experience (1–2 months) required between 5 and 10 minutes for each alignment per egg processed (Table 1). Operators with extensive experience (> 6 months) required 2 minutes for the first alignment (Step 20) and 1 minute for the second alignment (Step 22) for each egg processed (Table 1).

In addition to constraints in the SCNT cloning protocol, three quality management gaps were identified. The first was in Step 12, where collected eggs are placed in CSOF to prevent activation. The CSOF was collected from wild Chinook salmon. Laboratory members worked with state fisheries officials to secure 20–30 Chinook salmon every 2 years. Ovarian fluid collected from each fish was processed into individual batches of CSOF solution, and each batch was tested using *in vitro fertilization*. Eggs were placed in CSOF and fertilized after 1, 2, and 4 hours of incubation. Only CSOF batches with fertilization and survival rates above 70% (live embryos divided by total viable eggs) at 24 hours after-fertilization were used for SCNT. The use of CSOF in Step 12 constituted a quality management gap because other laboratories and facilities may be unable to collect and process salmon ovarian fluid every 2 years. In addition, processing the collected fluid into CSOF solution batches constitutes a system constraint because each batch requires circa 8 hours to process and test (pers. comm., W. Poulos and F. Jimenez).

Quality management gaps were also identified in Steps 11 and 26, when eggs and sperm were collected from zebrafish. In Step 11, the quality of the collected eggs was not always apparent to less-experienced laboratory members. This could lead to wasted work effort in the remainder of the SCNT cloning protocol if poor-quality eggs were processed, resulting in a low number of viable embryos. Similarly, in Step 26, the quality of collected sperm was not assessed before fertilization in Step 27. This could result in poor fertilization and lead operators to believe that egg quality or culturing conditions were poor.

## Discussion

### Operator experience

The effective progression of a research protocol, such as SCNT cloning, toward a reproducible community-level pathway is crucial for ensuring methodologies can be replicated across multiple facilities. To achieve this objective, we outlined the steps of the cloning protocol in a process map, identifying constraints and the requisite training levels for each step. Most tasks in the SCNT cloning pathway can be executed by Part-Time laboratory members, who can complete training within two months. However, Steps 17–23 demanded over four months of training and are exclusively reserved for Long-Term laboratory members. Clearly defining which steps can be performed by different personnel is vital for efficient processing.

The concept of "production leveling" from industrial engineering describes production schedules that are designed to maintain continuous workflow and distribute workloads evenly throughout the day [30]. Assigning less training-intensive tasks to Part-Time members enables Long-Term members to focus on the more skill-intensive steps without causing production bottlenecks. It is essential to recognize that training all laboratory members for Steps 17–23 may not necessarily enhance workflow efficiency. Training costs, encompassing both direct and indirect costs [18] should be weighed against the benefits. Training should only be pursued when the expected benefits exceed the incurred costs. For instance, training Part-Time members, such as student workers, for Steps 17–23 might be unnecessary if they do not plan to work in the laboratory for an extended period or if it hinders progress in other pathway steps. Furthermore, relying on Part-Time members or beginner operators to process embryos in Steps 17–23 would decrease production capacity, as observed during simulation modeling. Only operators with expert skill ratings would be able to process the required number of embryos (30 embryos on 6 manipulation dishes) within the 2-hour time limit before embryos quality starts to decrease (Fig 3).

The training of operators in advanced skills, like micromanipulation in Steps 20–23, demands significant time and resources. Laboratories initiating SCNT should consider hiring experienced personnel to supervise and mentor less experienced colleagues. Competence in cell culture procedures, encompassing media composition, preparation, utilization, storage, and skills in micromanipulation systems, microscope-mounted lasers, electroporation, and micro-pipetting, is essential for personnel engaged in somatic cell nuclear transfer.

### Protocol constraints and quality management

In addition to specifying training requirements, another critical element in advancing a protocol towards a reproducible pathway was the identification of protocol constraints, accompanied by recommendations to address them. The dechorionation of 24-hour post-fertilization embryos emerged as a significant time constraint in the SCNT cloning pathway. Manual removal of individual chorions using two hypodermic needles, while effective, proved time-intensive, especially for novice technicians. This bottleneck was unexpected, as during initial discussions with laboratory personnel no steps in the Cell Culture Stage were thought to be time constraints. Previous studies have also identified unexpected bottlenecks, particularly when one operator must process all samples individually to complete a step before continuing the process [17]. Potential solutions involved adding additional operators to assist with the constraining step or modifying the step so a single operator could process samples more quickly. In this study, a brief incubation (1 min) in 2 mg/ml pronase (Cat no. 10165921001, Roche) and Embryo Medium at 28.5°C decreased the time required to dechorionated each embryo. Following bulk pronase treatment of egg clutches, rinsing embryos in fresh media three to four times facilitated chorion removal. Pronase treatment, if properly executed, drastically reduced processing time and increased bulk embryo throughput. However, prolonged exposure to pronase could degrade embryos, leading to cell death before plating. To mitigate this, we recommend thorough training and observation of proficiency in embryo manipulation, particularly for novice individuals, before pronase treatment.

Beyond biological approaches, the development of engineered devices for egg manipulation could enhance efficiency. Automated technology options for steps that currently require high levels of training and manual skill would reduce human error, and would improve precision and reproducibility. In Steps 20–23, where micromanipulation of eggs is both skill and time-intensive, the use of microfluidic devices to control egg orientation shows promise. Such devices, as reported in zebrafish embryo manipulation tools, could be produced through 3-D printing for wider distribution as open hardware [31, 32]. It is important to note that the non-spherical nature of unfertilized zebrafish eggs presents a challenge when developing microfluidic manipulation devices. However, with the increasing capability of 3-D printing for microfabrication [33, 34], egg manipulation devices could be designed and distributed as open hardware (as in technology that is available to distribute and modify freely). This promotes community-level sharing and improvement [35, 36].

While overcoming major constraints enhances production capacity, integrating quality management into the pathway is crucial for ensuring consistent results across facilities. A notable quality management gap in the SCNT cloning pathway was the use of CSOF in Step 12 to prevent egg activation. As interest in SCNT cloning in zebrafish grows and as the technique is adopted in more facilities, CSOF may become commercially available. However, CSOF is not sold commercially to date and collecting and preparing ovarian fluid from wild salmon is impractical for many facilities. Therefore, alternative solutions need to be considered to facilitate wider participation without compromising sample quality (a concept called harmonization is discussed in greater detail in Section 4.3). Osmolality studies on zebrafish sperm motility suggest that Hanks’ Balanced Salt Solution (HBSS) could offer an alternative to CSOF as sperm motility is completely inhibited at osmolalities greater than 300 mOsmol/kg [37]. Future research on the effect of osmolality and various ions on zebrafish egg activation could result in an alternative solution to CSOF using HBSS, which is commonly used in cryopreservation and can be prepared and modified “in-house” [37, 38].

Other quality management gaps were identified in Steps 11 and 26, which involved collecting germplasm (eggs or sperm) from zebrafish. Assessing the initial quality of germplasm immediately after collection is a recommended quality control step to prevent “defects” (low-quality samples) further down the production stream [29, 39, 40]. If initial germplasm quality is low, samples can be discarded, and new germplasm of higher quality can be collected to prevent wasted effort (i.e., a process that results in low cloned embryo survival). Measuring sperm motility is a common method of evaluating sperm quality; higher motility indicates higher quality sperm [37, 40, 41].

The equipment needed to measure sperm motility can be found in most research facilities, requiring a regular phase-contrast microscope and a counting chamber, such as a hemocytometer. To start, sperm motility should be recorded after collection and at other specific times [42] to evaluate quality and help explain abnormal results, such as if control IVF survival is abnormally low. Additionally, future studies could determine the minimum levels of sperm motility required to achieve consistent embryo quality. This type of quality assurance check has already been implemented in the pathway where embryo fertilization must be above a certain threshold to proceed (after Steps 2, 9, and 28). The quality of eggs collected could also be assessed using an egg quality diagram, and low-quality eggs could be discarded before they move further down the production line. Viable eggs are slightly granular, yellowish in color, have a “full” look, and the chorions can be observed to elevate away from the plasma membrane when ovarian fluid is diluted with water [43].

Implementing a unified quality management system across facilities requires community formation and collaboration. An active zebrafish research community already exists, with ongoing work to advance methodologies. Groups within the zebrafish community, such as ZIRC or the International Zebrafish Society, can lead efforts to implement quality management systems. These groups can hold workshops at conferences to gather input from community members. They could also employ process mapping and partial budgeting tools to identify gaps in quality management and solutions that can be sustainably integrated into zebrafish research and storage pathways. Genetic stock centers such as ZIRC could encourage quality control checkpoints by requiring certain data (i.e., sperm concentration, sperm motility) to be reported before samples are imported and stored in the facility. Publishing papers or reviews in widely read zebrafish journals would also raise awareness of the necessity of quality management.

### Creating a generalizable pathway

A final and crucial activity of developing a reproducible pathway from a research protocol, involves harmonization and generalization. The SCNT cloning pathway illustrated in Fig 2 was specifically generated to showcase the process in the Cibelli laboratory. While this process map is beneficial for analyzing constraints and quality management limitations within a particular facility, its direct application to other facilities may pose challenges due to potential variations in specific steps. For instance, research laboratories with prior experience in cell culture may employ different methods or equipment in Steps 1–7 for collecting, dissociating, and cryopreserving cells. Despite these differences, the goal is to ensure that the SCNT cloning pathway remains harmonizable and generalizable, maintaining consistent quality across facilities regardless of methodological disparities or equipment distinctions.

Harmonized pathways allow for flexibility in the nuances of steps, such as the collection of eggs (Step 1) or the choice of devices for cell freezing (Step 7), without compromising the quality of the final product [44, 45]. This is in contrast to standardization, where all facilities would be mandated to adopt identical equipment and methodologies, which may be an impractical expectation considering budget and training (i.e., effort) constraints [45]. Thus, harmonized pathways will allow a greater number of facilities to participate. To ensure harmonization, quality management and data reporting steps must be integrated into the pathway. Examples of this already exist in the SCNT cloning pathway outlined in Fig 2, where at several points the cloning process is terminated if fertilization rates do not meet minimum standards. These quality control checkpoints prevent low-quality cells from passing through the system, which would produce low-quality embryos. By implementing additional quality management checkpoints into the pathway, such as requiring sperm motility data to be recorded after Step 26, many facilities could participate in SCNT cloning and produce cloned embryos of consistent quality.

**Fig 4:**
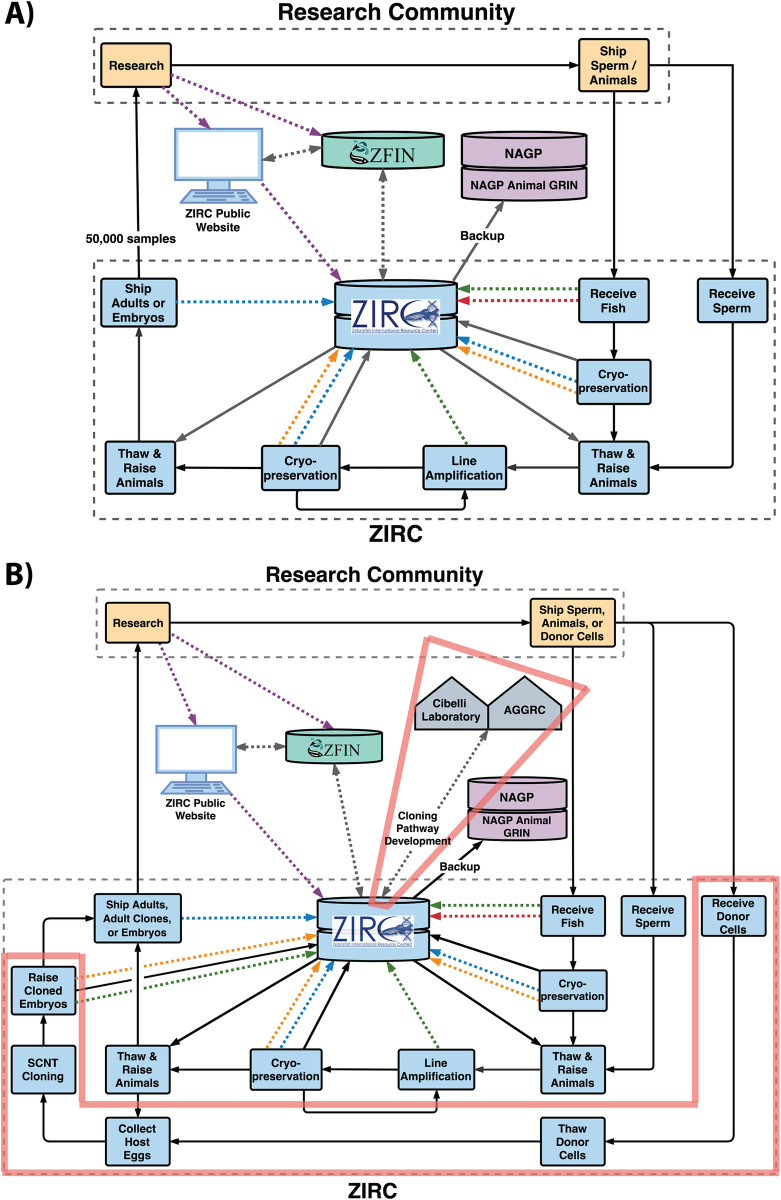
Center-level diagram of the Zebrafish International Resource Center (ZIRC). This diagram displays ZIRC before (A) and after (B) future integration of the SCNT cloning pathway (new steps and facilities are outlined in red). The diagram shows the interactions of ZIRC (in blue) with the research community (in yellow), the Zebrafish Information Network (ZFIN, in teal), pathway development collaborators (Cibelli laboratory and the AGGRC, in gray), and the USDA National Animal Germplasm Program (NAGP) repository and database (in purple). Solid black arrows indicate the transfer of physical material and dashed arrows indicate the flow of information (red indicates the transfer of Genetic Data, blue indicates the transfer of Cryopreservation Data, orange indicates the transfer of Sample Data, green indicates the transfer of Biological Data, and gray indicates multiple types of information).

Not all facilities will participate in every step of the SCNT cloning pathway. For example, as a genetic stock center, ZIRC may opt not to culture and cryopreserve cells (Steps 1–7) but instead receive and store frozen cells (to be used for nuclei donation) sent from their research community. When laboratories order cloned embryos, ZIRC personnel can enter the SCNT pathway at Step 8, continue down the pathway to produce clones and ship them to the laboratory (Fig 4). Likewise, members of the zebrafish community can conduct research with cloned embryos without every member needing to participate in the nuclear-transfer process (Steps 8–30). Harmonization is crucial for members of a research community to exchange genetic resources while maintaining quality. Other facilities, such as the USDA National Animal Germplasm Program (NAGP), may only engage as an additional backup facility for storing physical samples and data. The participation of a federal facility could also direct the development of quality management guidelines to ensure pathway harmonization.

Generalizability is another critical aspect integrated into the zebrafish SCNT cloning pathway. The National Institutes of Health, Office for Research Infrastructure Programs (NIH ORIP) recognizes zebrafish as a vital biomedical model and acknowledges three additional vertebrate and invertebrate aquatic models. Developing separate SCNT cloning protocols for each species would be impractical in terms of time, labor, and cost [46, 47]. Much like cryopreservation research, generalizing the SCNT cloning pathway efficiently expands the technique to other biomedical model species [40, 48]. The SCNT pathway outlined in Fig 2 can be generalized into nine major steps: 1) Collect Donor Embryos, 2) Prepare and Dissociate Donor Embryos, 3) Cryopreserve Donor Cells, 4) Collect Host Eggs, 5) Prepare Host Eggs for Cloning, 6) Thaw Donor Cells, 7) Prepare Cloning Equipment, 8) Enucleate Host Eggs and Transfer Nuclei, and 9) Raise Cloned Embryos.

This approach allows discoveries made during the development of a new protocol to apply universally to all SCNT pathways, streamlining efforts and promoting efficiency. ZIRC’s experience in harmonizing pathways for cryopreservation across multiple aquatic biomedical species demonstrates the potential for this strategy. By taking the initial steps to harmonize and generalize the SCNT cloning pathway, this technology can be systematically implemented at multiple zebrafish research facilities and also extended to other aquatic biomedical models, such as *Ambystoma mexicanum* [49] and *Xenopus laevis* [50]. It should be noted that while some major steps of the SCNT pathway can be generalized for new species, specific aspects of the steps may not be directly transferrable among species. For example, dechorionation of embryos, a major challenge identified in this study, may not be necessary in other species. A generalizable SCNT pathway will be flexible enough to incorporate additional steps or eliminate unnecessary ones. Pairing SCNT cloning with repository storage, for example, would allow facilities to preserve diploid genomes long-term. Cryopreserving and storing a portion of diploid somatic cells would safeguard diploid genomes, prevent wasted effort, and make genomes easily transferable between facilities.

## Conclusions

This study is, in many ways, a bridge between a constrained past and a diversified future approach. The safeguarding and dissemination of aquatic genetic resources, particularly for biomedical models, holds immense significance. Yet, the development of repositories for aquatic species lags considerably behind traditional crops and livestock in various aspects. This poses a substantial challenge given the global importance of aquatic species in providing food, livelihoods, as well as screening for cures and developing treatments for human diseases and conditions. Moreover, it undermines past and future investments in genetic management, as the inability to preserve valuable genetics in any form other than live populations or haploid genomes hampers progress [45]. Cryopreservation and repository storage of diploid somatic cells and haploid germplasm cells are each key to safeguarding valuable genetic resources for conservation and future research. Challenges with repository development, persistent for decades, have gained urgency due to the rapid advancement of genetic manipulation techniques for biomedical species. Rapidly developing SCNT technology has led to a surge in new lines and varieties, creating a pressing need for their preservation [51]. The reliance on traditional laboratory research by individual groups has proven inadequate for developing applied repository capabilities to address these large-scale challenges. Addressing such complex issues necessitates a community-level approach, utilizing new tools and interdisciplinary strategies focused on establishing applied capabilities across various scales [15, 17, 45]. These tools enable the visualization of interactions among technology development centers, stock centers, user communities, and other entities, whether private or federal.

In this context, industrial engineering tools such as process mapping, are highly useful in a biological context to create a community-level forum (pathway). This pathway serves to integrate and align the efforts of multiple groups that would otherwise be in competition while working toward divergent goals. Moreover, when properly designed, process mapping can support comprehensive simulation modeling—providing powerful analysis and optimization of real-world factors such as time, cost, personnel, and quality at facility, community, and network levels. These advancements can be further enhanced at the community level by incorporating open hardware—devices and technologies that can be shared as digital files and fabricated by users. Processing mapping can also guide future economic analysis to ensure implementing SCNT technology into a facility is cost-effective and sustainable. Interdisciplinary approaches are essential, and efforts are already in progress, especially as fabrication tools like 3-D printers become more affordable and widespread. For instance, to apply an interdisciplinary approach, biology laboratory groups could enlist engineering undergraduates, alongside their usual biology students, to gain access to the tools described above. Establishing outward-facing interdisciplinary technology centers to support community-level repository development would also significantly contribute to this collective effort.

## Supporting information

S1 File (DOCX)

S1 Graphical abstract (PNG)
