# Supplementary material for: Advancing nuclear transfer cloning in zebrafish (Danio rerio) into a translational pathway using interdisciplinary tools
Source: PLoS One. 2024 Dec 30;19(12):e0312672. doi: 10.1371/journal.pone.0312672 (PMC11684642; doi:10.1371/journal.pone.0312672)
Supplement: S1 File — (DOCX) [file pone.0312672.s001.docx]

**Supplementary Material**

*Embryo Media Solution*

Embryo medium was prepared as described in Westerfield (2000). Hanks’ balanced salt solution (HBSS, 271 - 300 mOsmol/kg, Cat no. H1387, Sigma) was used as a 10X stock. The stock was filtered with a bottle top-filter (unit 0.22 mm, Cat no. 431098, Corning) and kept at 4°C. Before use, the 10X stock was diluted with distilled water to 27.1 - 30.0 mOsmol/kg and kept at room temperature. The pH was also adjusted to 7.2 using drops of 1 M NaOH and a pH meter (Pinnacle 530 pH meter - M 530P, Corning).

*DNAC Culture Media*

The base media, DMEM (Gibco, cat# 11965092), was supplemented with N-Acetyl-L-cysteine (NAC) (Sigma-Aldrich, cat#A9165) to create DNAC culture media. In addition, Ascorbate-2-phosphate (A2P) (Dissolve in ddH2O, Sigma-Aldrich, cat#A8960), 100X Anti-Anti (Thermo Fisher Scientific, cat#15240-062), Bovine insulin (Cell Application INC, cat#128-100), Trout serum (SeaGrow®, East Coast Biologicals), and Fetal Bovine Serum (Thermo Fisher Scientific, cat#10439) were added to complete the DNAC culture media.

*DNAC Freezing Media*

Using the DNAC Culture media list above, 1 mL DNAC media was combined with 3 additional mL of Fetal Bovine Serum and 1 mL DMSO (Dimethyl Sulfoxide (J.T. Baker, cat#9224-01)). This solution is diluted with 4 mL of DNAC (9mL total volume) before being added as a cryoprotectant to cells before freezing. It should be noted that DMSO is toxic to the cells at room temperature, therefore, the exposure time before freezing is limited (e.g., the equilibration time).
