# Supplementary figures and images for: Advancing nuclear transfer cloning in zebrafish (Danio rerio) into a translational pathway using interdisciplinary tools
Source: PLoS One. 2024 Dec 30;19(12):e0312672. doi: 10.1371/journal.pone.0312672 (PMC11684642; doi:10.1371/journal.pone.0312672)

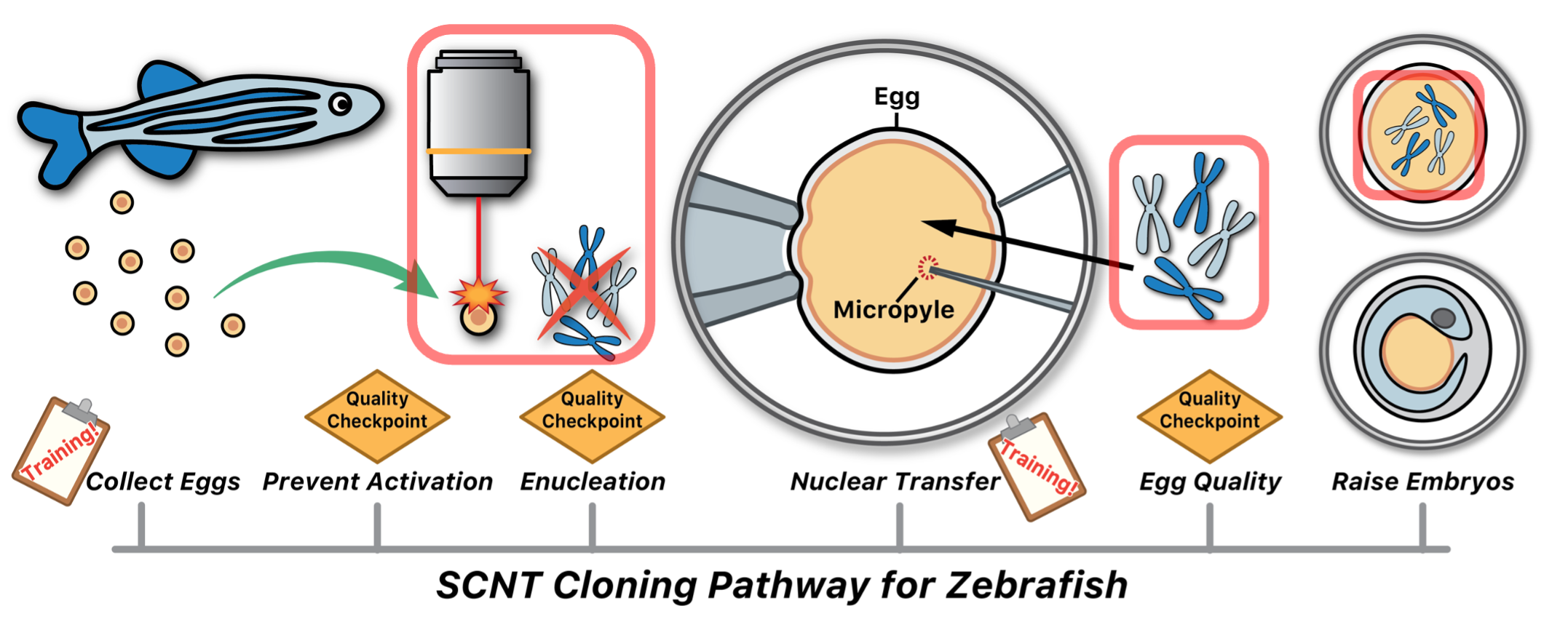

Supplement: S1 Graphical abstract — (PNG) [file pone.0312672.s002.png]
